# Subsurface Microbial Hydrogen Cycling: Natural Occurrence and Implications for Industry

**DOI:** 10.3390/microorganisms7020053

**Published:** 2019-02-15

**Authors:** Simon P. Gregory, Megan J. Barnett, Lorraine P. Field, Antoni E. Milodowski

**Affiliations:** British Geological Survey, Environmental Science Centre, Keyworth, Nottingham NG12 5GG, UK; lorfie@bgs.ac.uk (L.P.F.); aem@bgs.ac.uk (A.E.M.)

**Keywords:** hydrogen, hydrogenotrophy, corrosion, gas storage

## Abstract

Hydrogen is a key energy source for subsurface microbial processes, particularly in subsurface environments with limited alternative electron donors, and environments that are not well connected to the surface. In addition to consumption of hydrogen, microbial processes such as fermentation and nitrogen fixation produce hydrogen. Hydrogen is also produced by a number of abiotic processes including radiolysis, serpentinization, graphitization, and cataclasis of silicate minerals. Both biotic and abiotically generated hydrogen may become available for consumption by microorganisms, but biotic production and consumption are usually tightly coupled. Understanding the microbiology of hydrogen cycling is relevant to subsurface engineered environments where hydrogen-cycling microorganisms are implicated in gas consumption and production and corrosion in a number of industries including carbon capture and storage, energy gas storage, and radioactive waste disposal. The same hydrogen-cycling microorganisms and processes are important in natural sites with elevated hydrogen and can provide insights into early life on Earth and life on other planets. This review draws together what is known about microbiology in natural environments with elevated hydrogen, and highlights where similar microbial populations could be of relevance to subsurface industry.

## 1. Introduction

Historically, there has been a lack of interest in the presence of hydrogen in the subsurface due to a misconception that it does not occur freely [1]. It is now recognized that hydrogen gas occurs in a wide variety of geological settings and derives from several different processes [1,2]. With this recognition, there is a growing interest in the possible use of geological hydrogen as a fuel source and in the use of suitable geological formations for hydrogen storage. The main hydrogen occurrences are associated with graphite deposits, volcanic systems, geothermal systems, ultramafic rocks, alkaline igneous complexes, crystalline basement, potash and evaporite deposits, cataclasites, and anoxic sediments. Anomalously high occurrences of hydrogen are also associated with oil and gas fields, as well as coals. An increasing awareness that biological processes are coupled to other thermal, hydraulic, mechanical and chemical processes has led to an appreciation of the importance of hydrogen-metabolizing microorganisms in the subsurface. The wide variety of environments where hydrogen is produced or accumulates might also provide insights into processes, including microbial metabolisms in, often difficult to access, engineered subsurface environments. The deep subsurface can be inhospitable for microorganisms, with combinations of extreme temperature, limited nutrient availability and limited energy sources. Furthermore, the availability of void space often constrains microbial activity as cells require pore networks with spaces large enough to pass through [3,4,5]. Despite these limitations, microorganisms persist to great depths [6,7], and hotspots of activity may occur under appropriate physical conditions where energy can be obtained from redox reactions involving electron donors such as methane, iron, or hydrogen [8,9,10]. Hydrogen gas has a low reduction potential and is, thus, a highly energetic electron donor when involved in sulfate, carbon dioxide, and ferric iron reduction, among other processes. These processes may compete against each other for a limited supply of hydrogen, particularly in oligotrophic deep-subsurface environments [11].

In the natural environment, hydrogen is produced and consumed by both abiotic and biotic processes. Subsurface environments where hydrogen production takes place can host diverse and active microbial communities, when other environmental conditions (e.g., water, space, electron acceptors, temperature) allow. In engineered subsurface environments, hydrogen can be artificially elevated either through the storage of hydrogen (for use as a fuel), as an impurity of storage of other gases, or by hydrogen generation through corrosion of steel infrastructure. This hydrogen is a potential source of energy for microbial processes in engineered environments and has implications for corrosion and gas production and consumption. Understanding microbiological processes that produce or consume hydrogen not only informs processes relevant to industry, but can also inform our understanding of natural processes, the development of life on Earth, and exobiology. Due to the widespread nature of microbial processes involving hydrogen, and its implications for a wide range of disciplines, it is timely to review our current knowledge and understanding of these processes. 

In order to standardize the values in this manuscript, the units of some original data have been converted. All gas volumes are converted to moles assuming standard conditions, and all energetic calculations are relative to one mole of hydrogen.

## 2. Hydrogen Generation and Consumption in Subsurface Environments

In the subsurface, both biotic and abiotic processes can generate and consume hydrogen, though the relative contributions of different processes are often not well assessed. Abiotic processes can operate over a wider range of temperatures (to in excess of 600 °C), but biotic processes are limited to cooler environments where conditions are more favorable for life. Where abiotic hydrogen generation occurs at high temperatures, or involves radiation, this may inhibit microbial life in the immediate vicinity. This hydrogen could become available for microorganisms if it migrates away from the source of heat or radiation. In these environments, hydrogenotropic communities are likely to develop and contribute to hydrogen removal. 

## 3. Abiotic Hydrogen Generation

Several processes are responsible for abiotic hydrogen generation within the Earth’s crust (Table 1). These include the following:

- Graphitization (the decomposition of methane to graphite). This potentially occurs at temperatures above 600 °C, though limited information is available on natural occurrences in the geosphere [1,12].
- Radiolysis of water (the dissociation of water molecules by ionizing radiation from radioactive decay). The main naturally occurring radioactive elements are uranium, thorium, and potassium. This results in the production of H^•^ and OH^•^ radicals (and the subsequent formation of H_2_ and H_2_O_2_). α- and β-radioactivity are probably the most efficient in producing hydrogen, with production from α-radiation greater than β-radiation [1,12,13].
- Reaction of ^40^Ca (produced by the radioactive decay of potassium) with water forming calcium hydroxide and hydrogen [1,14,15,16].
- Serpentinization (the process whereby primary, high-temperature igneous ferromagnesium minerals are altered to low-temperature, secondary serpentine minerals through interaction with low-temperature water percolating through these rocks). This typically occurs below 500 °C, with peak rates between 300 °C and 390 °C, but can occur more slowly below 100 °C. At elevated pressure (35 MPa), peak production occurs at 200–315 °C [12,17,18,19,20].
- Cataclasis (hydrogen generation in fault zones by several different processes). Hydromechanical reactions break chemical bonds and form radicals that react with groundwater to produce hydrogen. Reduction of water by reaction of ferrous iron minerals contained in silicate minerals in crushed rock may be another potential mechanism of hydrogen release in this scenario. Up to 0.22 moles of hydrogen produced per m^3^ of andesite has been reported by this mechanism [21,22,23,24].
- Reactions between dissolved magmatic gases (degassing of primordial hydrogen from deep mantle or hydrogen reintroduced during subduction) [25].

The contributions of each of these processes to the gaseous composition of the subsurface will depend on the environment, and the relative importance of different processes is yet to be assessed.

## 4. Microbial Hydrogen Generation

Microorganisms are capable of producing hydrogen through fermentation, nitrogen fixation, anaerobic carbon monoxide oxidation, and phosphite oxidation [26,27]. The enzyme hydrogenase is generally responsible for the production of hydrogen in these processes by catalyzing the reaction as follows:

H_2 (g)_**⇆** H^−^ + H^+^**⇆** 2H^+^ + 2e^−^ (1)

Hydrogenases are divided into three different types, depending on the metal in the active site: [FeFe], [NiFe], and [Fe]. Hydrogen generation is generally associated with [FeFe]-hydrogenases and these are typically found in anaerobic Bacteria and some Eukaryotes; they have not yet been found in Cyanobacteria or Archaea. Some groups of [FeNi]-hydrogenases are also implicated in hydrogen generation and these are found in Cyanobacteria and Archaea. [FeNi]-hydrogenases are also found in a large number of Bacteria [28]. In recent years, much has been learnt from research into the use of microorganisms for hydrogen production as an industrial feedstock or energy carrier [29,30]. This technology usually relies on hydrogen production by photosynthetic organisms (which is not relevant to the subsurface), fermentation of organic compounds, or microbial electrolysis cells. Many of the organisms used in fermentative technology are common in subsurface environments, e.g., *Bacillus*, *Clostridium*, and *Desulfovibrio* species. Another mechanism, hydrogen production from phosphite oxidation (Table 2), has been reported in the laboratory only, in *Escherichia coli* under anaerobic conditions [31]. Although there is a growing body of literature on these hydrogen-producing processes in the laboratory or when applied to hydrogen production for energy, much less is known about similar processes occurring in the natural environment or in engineered subsurface environments. Fermentation of organic matter will be important in subsurface environments where there is sufficient organic carbon (either naturally occurring or introduced, e.g., by pollution). Conversely, in other environments, such as crystalline basins, fermentation is likely to be less significant, due to limited organic carbon sources [7]. End-product inhibition of fermentation necessitates that the hydrogen production must be balanced by hydrogen removal. Therefore, hydrogen, as a product of fermentation, is normally a transient substance, being rapidly utilized as an electron donor. In aquatic subsurface environments (sediments and aquifers), hydrogen concentration varies in response to the dominating terminal electron acceptor process (TEAP), i.e., hydrogen-consuming process. Each terminal electron acceptor reaction has a characteristic minimum steady-state hydrogen concentration associated with it (hydrogen thresholds are shown in Table 3).

In addition to hydrogenases, nitrogenases are capable of producing hydrogen gas as a by-product of nitrogen fixation [32]. Nitrogen fixation occurs in both oxic and anoxic environments [26] and converts one mole of nitrogen gas into two moles of ammonia and one mole of hydrogen (Table 2). The ability to fix nitrogen is widespread amongst prokaryotes but is energetically expensive, requiring 16 molecules of ATP to fix one molecule of dinitrogen gas [33]. Nitrogen fixation is little studied outside of the rhizosphere, but evidence for it has been reported in the subsurface, e.g., in sulfidic sediments in cave/mine environments [34,35,36], hydrothermal vents in the Juan de Fuca ridge [37], deep terrestrial environments [38]. and in anoxic methane seeps in mud volcanoes in the Kumano Basin, Japan [39]. Nitrogen-fixing genes are present in the genomes of many anaerobes typical of subsurface environments (including methanogens, acetogens, fermenters, and sulfate reducers), although the true diversity, distribution, and activity of nitrogen fixation remains uncertain [40]. Of course, it should be remembered that the detection of a particular functional gene in an environment does not necessarily mean that microorganisms are actively carrying out that function.

## 5. Abiotic Hydrogen Consumption

Abiotic hydrogen consumption typically occurs at high temperatures (well above those where microbial processes can operate), during magma cooling and in hydrothermal systems through the reduction of carbon dioxide to form methane and other hydrocarbons [44]. However, there are processes that can naturally occur within microbial temperature ranges, such as low-temperature methanation through the Sabatier reaction, or Fisher–Tropsch-type synthesis, e.g., in the Chimaera seep in Turkey [45], where the reactions occur at temperatures below 50 °C. In the presence of chromium mineral catalysts, this could convert significant amounts of hydrogen to methane over geological time scales [46]. Although not technically consumption of hydrogen, a recent study found that sorption of hydrogen to clay can be a significant hydrogen sink [47].

## 6. Microbial Hydrogen Consumption

Hydrogen-based metabolisms are thought to have been critical to life on early Earth; however, with the advent of oxygenic photosynthesis, hydrogen-based life became confined to specialized niches [26]. Hydrogen appears to be the main source of energy for autotrophs in many subsurface environments, especially those that have no, or a limited supply of carbon from photosynthesis [48]. Even at low concentrations, various microbial metabolisms can be supported by hydrogen when a suitable terminal electron acceptor is available (Table 3). These metabolisms include methanogenesis, acetogenesis, sulfate-, sulfur- and iron (III) - reduction, aerobic hydrogen oxidation, dehalorespiration, fumarate respiration, denitrification, and of less relevance to the subsurface, anoxygenic photosynthesis [26,49]. The most important microbial hydrogen-consuming processes are shown in Table 3. An interesting addition to this list comes from the recent finding that lithoautotrophic Cyanobacteria were the most dominant taxa in microbial communities in the Rio Tinto Pyrite Belt, Spain [50]. Cyanobacterial abundance was inversely correlated with hydrogen abundance, and modeling suggested that the abundance of the cyanobacterial families Rivulareaceae and Xenococcaceae, along with Sphingomonadaceae and Bradyrhizobiaceae, best explained the hydrogen abundance pattern. This, combined with metagenomics data, led the authors to hypothesize that these taxa are responsible for the consumption of hydrogen and obtain their energy by coupling hydrogen oxidation to the reduction of a range of electron acceptors. Although cyanobacteria have previously been reported in various deep environments, their abundance has been difficult to explain [51,52,53,54]. This latest discovery at Rio Tinto suggests that these non-photosynthesizing Cyanobacteria might be active members of the microbial community, rather than inactive cells that were washed down and trapped in the subsurface or arising from contamination during sampling, as previously postulated.

The hydrogenase reaction discussed in Section 4 is reversible and is involved in coupling hydrogen uptake to the reduction of electron acceptors (e.g., nitrate, sulfate, and carbon dioxide). It has been hypothesized that high concentrations of hydrogen select for a microbial community containing species in possession of low-affinity variants of [NiFe]-hydrogenases [55]. [NiFe]-hydrogenases are widespread throughout the bacterial and archaeal kingdoms (including typical subsurface anaerobic organisms such as sulfate reducers, iron reducers, acetogens, methanogens, and fermenters [56]). Hydrogenase gene abundances in subsurface environments, per megabase, are nearly an order of magnitude higher than for surface environments [57], highlighting the importance of hydrogen cycling in many subsurface microbial communities.

Subsurface lithoautotrophic microbial ecosystems (SLiMEs) are particularly interesting ecosystems, and are defined as those which are entirely independent of photosynthesis for carbon and hydrogen supply. Such systems are driven by hydrogenotrophy and are of interest because of their relevance to early Earth history and the search for life on other planets [62]. Examples of systems suggested to be reliant on hydrogen as an energy source include methanogen-dominated communities in hydrothermal waters circulating through igneous rocks at Lidy Hot Springs, Idaho [8], environments beneath deep-sea hydrothermal fields in the Central Indian Ocean Ridge [63], deep basaltic aquifers [7,64], and marine sediments [65]. In these ecosystems, it is proposed that energy is obtained by coupling hydrogen oxidation to reduction of electron acceptors such as carbon dioxide, nitrate, or ferric iron, although it has proven difficult to demonstrate that there is definitely no flow of elements from present day, or past photosynthesis. Calculations suggest that these low-biomass and low-diversity microbial communities can be supported by observed rates of radiolytically produced hydrogen [7]. The first claims of ecosystems that are independent of photosynthesis were made in Stevens and McKinley’s study of the Columbia River Basalt Group aquifer [64]. This study, which proposed a microbial ecosystem supported by hydrogen produced from the reaction of water with iron in ferromagnesian silicates, was controversial, and there was considerable debate around a number of issues including whether the amount of hydrogen experimentally generated from these rocks, at environmentally relevant pH, was sufficient to support the biomass; whether the rocks contain sufficient reduced iron to sustain microbial activity over geologically relevant timescales; whether or not organic material is more likely the source of hydrogen; and whether reduced gases from deeper in the Earth support the microbial ecosystem in this environment [66,67,68]. It will always be difficult to determine the contribution of different hydrogen-generating mechanisms in the deep subsurface; however, support for the existence of microbial ecosystems that are primarily, if not completely dependent on abiotic hydrogen sources in various geological settings is growing [48,62,63,69,70,71], including from some of the authors arguing against Stevens and McKinley’s original paper [8]. Independence from photosynthesis, including any contributions of carbon, nitrate, sulfate, and ferric iron, would be of great significance for the understanding of life on a pre-photosynthetic Earth and the possibilities of life on other planetary bodies, but remains to be unequivocally proven.

### Syntrophic Relationships

Biological production and consumption of hydrogen are usually tightly coupled through syntrophic relationships. For example, hydrogen that is produced by bacterial degradation of organic matter during the early (near-surface) diagenesis of sediments is usually quickly consumed; turnover of hydrogen may be high, but much of this hydrogen will be transferred between cells (interspecies hydrogen transfer) and never accumulates in the dissolved hydrogen pool [26]. In the past, the strength of some syntrophic relationships caused confusion; what is now known to be a syntrophic relationship between a hydrogenotrophic methanogen (*Methanobacterium bryantii*) and a bacterium that ferments ethanol to hydrogen and acetate was once thought to be a single species (*Methanobacterium omelianskii*) [72,73]. In most syntrophic reactions, a thermodynamically unfavorable (a positive standard free energy change (ΔG^0′^) fermentation reaction is coupled to a thermodynamically favorable (negative ΔG^0′^) hydrogenotrophic reaction. For example, the anaerobic oxidation of acetate by secondary fermenting syntrophic microorganisms, such as *Clostridium* spp., is an energetically unfavorable reaction (ΔG^0′^ = +26.15 kJ·mol^−1^ H_2_) and will only proceed if hydrogen-consuming microbial processes, such as hydrogenotrophic methanogenesis (ΔG^0′^ = −33.9 kJ·mol^−1^ H_2_), remove the hydrogen [74], and proceed under non-standard conditions. In this way, fermentation reactions that are endergonic can be made exergonic in situ if hydrogen is removed by hydrogen-consuming microorganisms [75,76]. 

## 7. Specific Environmental Settings: Relevant Hydrogen Sources and Hydrogen-Cycling Microorganisms

### 7.1. Subseafloor Environments

In deep anoxic ocean sediments, as the concentration of organic matter decreases, radiolysis of water seems to become increasingly significant as a source of hydrogen [77]. Based on the concentration of radioactive elements and the porosity and grain size of the sediment, Blair et al. [77] modeled the production of hydrogen through radiolysis, concluding that up to 10% of microbial respiration could be fueled by this hydrogen in such environments [62]. Dzaugis et al. (2016) suggested that, in young East Pacific oceanic basalts (<10 Ma), radiolytic hydrogen is a less important electron donor source than Fe (II) which is oxidizable. However, in contrast, radiolytic hydrogen in the older oceanic basalts may be the dominant electron donor source as little Fe (II) is accessible to oxidization and, therefore, serpentinization [78]. In that environment, it was calculated that radiolytic hydrogen production could support 30−1500 microbial cells in the volume of water adjacent to 1 cm^2^ of basalt, depending on its radioactivity. Hydrogen production rates of up to 4.8 × 10^−9^ mol H_2_ cm^−2^/year have been calculated for the sediments below the South Pacific Gyre [79]. Other studies suggested that abiotic hydrogen production alone is insufficient to support the observed microbial biomass in oceanic crusts and it has been hypothesized that fermentation by anaerobic fungi and other sources of organic material could provide additional sources of hydrogen in oceanic sediments [80]. 

In oceanic sediments, hydrogen can be biologically produced from a range of minerals at temperatures between 0 °C and 100 °C (particularly above 70 °C) [81], and biomechanochemical processes (stress and fracturing caused by tectonic activity, enhanced by microbial activity) are likely responsible for production of hydrogen in subseafloor sediments. Parkes et al. [81] indicated that, in this environment, the production of hydrogen is higher in the presence of prokaryotes than in abiotic controls, though the metabolic processes responsible for this were not clearly outlined. The hydrogen that is produced is required to support the observed amounts of biomass in these sediments. This hydrogen then further stimulates microbial sulfate reduction, acetogenesis, carbon dioxide production, and methanogenesis. More recent work demonstrated that hydrogen generated by crushing granite can support long-term methanogenic cultures [82]. Hydrogen released by comminution of rocks has also been shown to produce enough hydrogen to support methanogenesis in subglacial environments [83].

### 7.2. Hydrocarbon Reservoirs

Radiolysis may also be an important source of in situ hydrogen production in some hydrocarbon reservoirs. Typically, hydrocarbon reservoirs (e.g., North Sea reservoirs) are methane-dominated with hydrogen gas contents of less than 1% [1]. However, free hydrogen does sometimes accumulate, but the origin of hydrogen in these fields is often unknown. Radiolysis of water by the radioactive decay of potassium is a possible source since hydrogen tends to be more abundant where some of the reservoir or source rocks contain evaporite beds, particularly potash deposits [1]. In other cases, the hydrogen may have migrated from elsewhere, e.g., in the case of the Koksher gasfield, Estonia (nearly 21% hydrogen), gas may have migrated from offshore Swedish Baltic Cambrian fields [1]. Other processes that could account for the presence of hydrogen in hydrocarbon fields include thermal degradation of organic matter in sedimentary basins, serpentinization at depth, or biological aromatization of oil [84]. Evidence of hydrogenotrophic methanogenesis has been found in reservoirs from several deposits including Illinois Basin, United States of America (USA) [85], Woolaton Subgroup, Australia [86], and Southern Qinshui Basin, China [87]. Free hydrogen is rarely found in coals and coal mines, and the gas content in most coals is largely methane. However, anomalous coal-derived free hydrogen has been encountered in gases in some former Soviet Union mines, with 3–40% hydrogen [88]. Analysis of microbial communities in water associated with coal and oil deposits were found to be dominated by sequences related to hydrogenotrophic methanogens (Methanomicrobiales and Methanobacteriales) and a broad range of fermentative bacteria [89]. Mayumi et al. [90] showed, using experimentation and thermodynamic calculations, that increased carbon dioxide partial pressure, in microcosms simulating oil reservoirs, can increase the rate of methanogenesis due to a switch from syntrophic acetate oxidation and hydrogenotrophic methanogenesis to acetoclastic methanogenesis, which will have an impact on the dominating hydrogen-cycling process. Methanogenesis tends to increase at lower salinities [85], which may explain why certain shale plays, such as the Antrim Shale (Michigan Basin, USA) and Öland Island, produce methane with a significant biogenic proportion [91]. At both sites, dilution of salinity by glacial meltwaters is thought to have stimulated (hydrogenotrophic) methanogenesis.

### 7.3. Evaporite Deposits

Locally high concentrations of free hydrogen are sometimes associated with evaporite deposits, particularly potash, and may also be attributed to a combination of radiolysis of water and the subsequent reaction of water with ^40^Ca daughter product from the radioactive decay of potassium (^40^K) (e.g., References [14,15,16]). Notable occurrences of hydrogen in halite include the Leopoldshall Salt Mine in the Zechstein (Permian) halite deposits at Stassfurt in Germany, where hydrogen flowed for more than 4.5 years at a rate of 35.2 mol·day^−1^ [1]. In addition to radiolysis of water, hydrogen may originate from gas trapped during early biodegradation of organic matter within the halite [92], or may have migrated from elsewhere [1]. Although halite is a hostile environment for many microorganisms [93], viable Bacteria, and Archaea have been reported from fluid inclusions in evaporate minerals from deposits, e.g., Yunying Depression, China [94] and Saline Valley, USA [95]. Halotolerant Bacteria and Archaea have been shown to cycle hydrogen [96,97,98] and, although hydrogen has been detected within fluid inclusions [95], evidence for microbial hydrogen cycling is yet to be identified within such fluid inclusions. 

### 7.4. Crystalline Basement

Free hydrogen concentrations of up to 30% volume of the gas content have been reported from mines across the Canadian, Russian, and Fennoscandinavian shield areas and from boreholes in fractured Archaean crystalline basement such as the Pori and Juuka areas in Finland [1,99,100]. Much of this hydrogen is likely to be a result of radiolysis of water at low, near-surface temperatures, but active serpentinization might also account for most of the hydrogen encountered in the Juuka and Pori boreholes in the Fennoscandian shield [100]. Even higher hydrogen concentrations (68–88% total gas) were reported from boreholes in the granulite faces of the Kola Peninsula, close to the Finnish border, and at the Tarta dome borehole (96%). This may have been due to a dyke acting as a barrier to gas flow, causing hydrogen accumulation [14]. At the Äspö Hard Rock laboratory in Sweden (45 nM to 100 µM hydrogen in groundwater), located in fractured granitic rock, hydrogenotrophic methanogens and acetogens have been found, with acetogenesis supporting the activity of acetotrophic methanogens and possibly other acetate-utilizing organisms such as sulfate reducers and iron reducers [69,101]. Representatives of the *Hydrogenophaga* genus dominated the bacterial communities in groundwater in fractures in the granitic rocks in Olkiluoto, Finland when the sulfate concentration was low, and sulfate reducers (*Desulfobacula*, *Desulfovibrio*, *Desufobulbaceae*, *Desulfobacterium*, *Desulfosporosinus*, *and Desulfotignum*) dominated when sulfate concentration was high, although *Hydrogenophaga* species were still found in low abundance and vice versa [102]. At another site in Finland (the Outokumpu Deep Drillhole) with a range of hydrogen concentrations (0.008 mM to 3.1 mM), the environment is populated by bacterial species such as *Hydrogenophaga* spp. [103]. It was suggested that *Hydrogenophaga* could be occupying a low-hydrogen, low-oxygen niche at an oxic–anoxic transition zone. The archaeal community is dominated by hydrogenotrophic methanogens (Methanobacteriaceae) in the deep fracture zone. There is evidence for nitrogen fixation in crystalline basements [104], providing a potential additional source of hydrogen. Genomic data suggest that organisms with the genetic capacity for anaerobic hydrogen oxidation and nitrogen fixation seem to be involved in biofilm formation in the oligotrophic conditions at the Äspö Hard Rock Laboratory, and is an area deserving further research to understand microbial activity in these environments [35,104].

Fluids from Witwatersrand Basin, a large gold mining area of South Africa, contain hydrogen that is predominantly produced by radiolysis (potentially induced by the radioactive decay of uranium, thorium, and potassium). Measured hydrogen concentrations varied from 0.014 nM to 7.4 mM; however, the authors calculated much higher in situ hydrogen concentrations due to loss by abiogenic methane formation and diffusion, and the dissolved hydrogen concentration was sufficient to support the lithotrophic microbial communities present in the fracture water [105]. Genetic evidence from 16S ribosomal DNA (rDNA) and the methanogen-specific methyl coenzyme M reductase (*mcrA*) gene [106,107] suggests microbial involvement in hydrogen cycling within these groundwaters, with methanogens belonging to the Methanobacteriaceae identified at this site. Calculations suggest that the amount of hydrogen recorded in several Precambrian Shields is sufficient to support lithotrophic communities and inhibit hydrogen-producing fermentation taking place [71]. 

The Sanford Underground Research Facility, South Dakota, USA is located in metasedimentary rocks and accessed through a former gold mine. Studies of 16S rDNA sequencing revealed the presence of hydrogen-oxidizing, methanogenic, sulfate-reducing, and iron-reducing species, and metagenomic data suggest the ability to use hydrogen as an electron donor may be widespread amongst microorganisms in this environment [108]. Thermodynamic modeling showed that the in situ measured hydrogen concentration is too low to be a significant energy source; however, molecular data suggest a hydrogen-oxidizing community that is concentrated in biofilms. This may indicate an active microbial community utilizing the hydrogen as soon as it is produced [109]. 

### 7.5. Ultramafic and Mafic Rocks, and Geothermal and Volcanic Systems

Ultramafic rocks (e.g., ophiolite and layered igneous complexes), in particular, can be altered through the process of serpentinization [19,45,110]. Gas associated with these rocks is predominantly hydrogen, with lesser amounts of other gases, including nitrogen, oxygen, methane, and carbon dioxide [1]. Considerable seepage of hydrogen-rich gases as observed in association with serpentinites around the world (e.g., References [1,111,112]). Low-temperature serpentinization has also been invoked as the cause of abiotic gas production in the Zambales (Philippines) and Oman ophiolites [20,111,113]. The gas seep at Chimaera, Turkey is the largest in Europe, and one of the largest on-shore hydrogen seeps worldwide [114,115]. Here, methane-rich gases containing 7.5–11% hydrogen have been discharging for thousands of years [45]. Isotopic and other studies indicate that the released methane and hydrogen were formed by low-temperature (below 100 °C and below 50 °C, respectively) abiotic processes associated with serpentinization of the Tekirova ophiolite [45]. At the Chimaera site, isotopic and biomarker evidence points to additional active microbial methanogenesis (without any anaerobic methane oxidation) and subordinate sulfate reduction [116]. Near the surface at this site, a heterotrophic microbial community was characterized, and contained denitrifying bacteria but not nitrogen-fixing organisms [117]. 

Though uncommon, gas production from hyperalkaline calcium-hydroxide-rich waters issuing from serpentinized systems can have a low hydrogen content, e.g., the Othrys ophiolite [46], the Voltri Group [118], and at Genova, Italy [119]. This may be due to hydrogen consumption by carbon dioxide reduction (e.g., through abiotic Fischer–Tropsch-type synthesis or microbial methanogenesis [46]). 

Hydrogen gas has been reported from some alkaline igneous complexes, principally in carbonatite and nephelinite complexes, which include ultramafic rocks derived from the mantle [1]. The high alkalinity and low levels of electron acceptors in serpentinites present challenges to microbial life [120]. However, 16S rDNA in oxic groundwaters of the Leka ophiolite complex, Norway show bacterial communities dominated by close relatives of *Hydrogenophaga* spp. [121]. These organisms are chemoorganotrophic or chemolithoautotrophic, oxidizing hydrogen as an energy source, and utilizing carbon dioxide as a carbon source [122]. In this environment, it is not clear what process hydrogen oxidation is coupled to. Hydrogen produced at hydrothermal vent sites at oceanic ridges reaches concentrations of up to 15 mM in the discharging hydrothermal fluid [123]. Investigations at the Rainbow hydrothermal field located on the Mid Atlantic Ridge showed that hydrogen represents more than 40% of the total dissolved gas present in the hydrothermal fluid [124]. Microbiological investigations in these environments indicate that hydrogenotrophy is very important [125]. Brazelton et al. [126] found a high density of hydrogenases, suggesting a role for organisms belonging to Clostridiales in hydrogen production in serpentine-hosted, marine, and continental springs (Lost City hydrothermal field and Tablelands Ophiolite, Newfoundland, respectively). Their metagenomic dataset did not support the alternative hypothesis that Clostridia predominantly produce hydrogen via acetogenesis, but the authors conceded that further biochemical and physiological studies would be required to establish the role that Clostridia play in hydrogen production in this environment. The same study failed to identify significant genetic evidence of methanogens, but there is isotopic evidence of microbial methanogenesis occurring at other similar sites including the Lost City hydrothermal field [125]. Within the oceanic crust around Juan de Fuca Ridge, the source of hydrogen is both abiotic (iron oxidation by the dissociation of water) and biotic (fermentation), with large temporal and spatial variations in gas concentrations, indicating dynamic circulation with the potential for diverse microhabitats [127]. Isotope studies of the methane suggest both biotic and abiotic sources, and that the biotic methane is used as an energy source for methanotrophy. 

Serpentinization is also important as a source of hydrogen from geothermal and hydrothermal sites. Examples of significant hydrogen gas have been recorded in geothermal fluids at the Lardarello Geothermal Field in Italy (1.7–4.3% hydrogen in discharged gas), Lidy Hot springs in Idaho, Kerlingerfjoll and Landmannlauger hot springs in Iceland (9–36% hydrogen in discharged gas), and at Milos in Greece [1]. Hydrogen flux at Mt Erebus, Antarctica has been measured at 1.4 × 10^6^ mol·day^−1^ [128], and bacterial communities in sub-ice fumarolic vents contain hydrogen-oxidizing species, along with potential methanotrophs (that could be using the outgassed methane), without evidence of microbial methanogenesis [129]. Although hydrogen is known to be released from Mt Erebus, hydrogen was not measured for this study, so it may be that, at this location, methanogenesis was limited by hydrogen availability. However, potential hydrogen oxidizers, including *Thermocrinus ruber*, *Hydrogenobacter* spp., *Hydrogenobaculum* spp., *Hydrogenothermus* spp., and hydrogen-oxidizing sulfate reducers, dominate sequences from the Yellowstone geothermal ecosystem [130], and symbiotic relationships with hydrogenotrophic microorganisms may support entire ecosystems at hydrothermal sites [131]. 

### 7.6. Tectonically Active Areas

Mechanochemical interactions between fractured rock surfaces and groundwater can produce hydrogen in active fault zones such as Yamasaki Fault [132], Wenchuan Fault [133], and San Andreas fault [24]. Experimental studies subsequently demonstrated that hydrogen can be released during the crushing and grinding of rocks [22,23]. As discussed above, radiolysis and water–rock interaction can generate hydrogen, and tectonic processes resulting in cataclasis may accelerate hydrogen production by exposing fresh mineral surfaces to groundwater [134]. Serpentinization processes may also contribute to some of the hydrogen detected in some fault zones, e.g., San Andreas fault [135]. McMahon et al. [134] concluded that measured hydrogen and water contents in fault rocks were sufficient to support hydrogenotrophic microorganisms. DNA sequencing of microbial communities in springs with sources from deep fault zones shows a dominance of Archaea over Bacteria, with the community dominated by hydrogenotrophic methanogens belonging to the Methanobacteriales and Methanosarcinales orders [8]. Deuterium-enriched hydrogen is initially produced during earthquake faulting; however, hydrogen in natural faults is depleted in deuterium, and this could be a result of hydrogen-metabolizing organisms around natural fault zones, by catalyzing the hydrogen isotope exchange between gas and fluid phases [136].

## 8. Significance for Early Earth and Other Planetary Bodies

Exploration of some of the environments described above, particularly ophiolites, other ultramafic settings, and sites such as Rio Tinto in the Iberian Pyritic belt, suggested them as suitable analog sites to inform the search for evidence of life on early Earth and past or present life on other planets [137,138,139,140]. As described above, these sites are typified by hydrogenotrophic metabolisms. It has been suggested that submarine hydrothermal vents above serpentinites on early Earth provided suitable hydrogen gradients, as well as geochemical, temperature, and pH conditions, to be a candidate location for the origin of life on Earth [141]. In addition to hydrogen, this process will supply electron donors such as methane, formate, acetate, and ammonia, and essential elements such as transition metals and transition-metal sulfides [123]. Along with phosphate (dissolved in seawater), this process could supply all the elements essential for life. There has been much speculation about whether hydrogen-driven ecosystems could have evolved on Mars, and McMahon et al. [134] proposed that, even with the low levels of Martian seismicity, fault rocks contain sufficiently elevated quantities of hydrogen gas to support subsurface, spatiotemporally limited, hydrogenotrophy. Radiolysis and serpentinization in the Noarchian (3.7–4.1 Ga) Martian crust was likely to have produced similar amounts of hydrogen as the Earth’s Precambrian crust, which is known to support chemolithotrophic microbial life [142]. This study suggested that a potentially habitable environment existed on Mars from pre-Noarchian times and identified the subcryosphere highly fractured zone as having suitable temperatures for life. Post-Noarchian conditions became increasingly hostile to life, and present-day surface conditions present an oxidizing, highly irradiating, anoxic environment, making the subsurface, with its potential for liquid water, energy sources (e.g., hydrogen from serpentinization), and organic compounds, the most promising location to search for life [143]. Several extraterrestrial bodies are described as ocean worlds as they have, or may have had, oceans; therefore, they are the focus for the search for life beyond Mars, but within our solar system [144]. Saturn’s moon Titan has a thick atmosphere which contains 0.1% hydrogen [145]. McKay and Smith [146] postulated that there is potential for microbial methanogenesis from acetylene or ethane. Although Saturn’s ice-covered moon Enceladus does not have an atmosphere, recent evidence for molecular hydrogen in plumes has been found [147], and microbial methanogenesis has been demonstrated under predicted Enceladean physiochemical conditions [148].

## 9. Impact of Microbial Hydrogen Cycling on Subsurface Industry

In addition to understanding natural processes in different settings, understanding microbial consumption and production has relevance to a number of industries, where microbial activity could impact upon operation. Typically, these impacts are negative, e.g., leading to increased microbial-influenced corrosion or consumption of stored gases. The impacts on different aspects of subsurface industry are discussed in the sections below. 

### 9.1. Metal Corrosion in the Subsurface

Microbial-influenced corrosion is a concern for many subsurface industries, including hydrocarbon extraction and storage [149,150], radioactive waste disposal [151,152,153], and water distribution systems [154]. Microbially influenced corrosion is too large a topic to be adequately covered here, and the reader is directed to a review such as Reference [155] for a detailed overview. However, the relevance to this review is that metal corrosion (whether abiotic or microbially influenced) causes the release of hydrogen, and that this hydrogen can then become available for microorganisms to utilize. Hydrogen-consuming microorganisms including methanogens, sulfate reducers, and acetogens have been implicated in causing corrosion, as well as various classes of heterotrophic microorganisms. The two groups of microorganisms thought to be particularly important are sulfate reducers and methanogens [155]. Normally, in the presence of high sulfate concentrations, the sulfate reducers outcompete the methanogens due to their lower hydrogen threshold (see references within Reference [26]); however, methanogenesis can still occur in the presence of sulfate. Acetoclastic methanogens will also compete with sulfate reducers for acetate, but since methanogenesis can proceed through the degradation of methylated organic compounds (methylotrophic methanogenesis), methanogens will not always be in in direct competition with sulfate reducers for hydrogen [156]. 

### 9.2. Gas Storage

The subsurface can be used for the storage of a number of fuel gases (e.g., methane, hydrogen, or town gas) and for geological storage of carbon dioxide. Potential impacts of microbial activity include contamination of the stored gas by hydrogen sulfide, acceleration of corrosion by hydrogen sulfide production, precipitation of iron sulfide, biofilm formation, carbonate precipitation, or the production of methane (Netherlands Enterprise Agency, 2017). In underground gas storage in North Stavropol, the cultivable community was dominated by hydrogenotrophic methanogens, acetogens, and sulfate reducers [157,158]. In samples close to the edge of an underground methane store (90% methane, with C2–C5 alkanes) in the Upper Jurassic Calcareous, Paris Basin, abundant DNA sequences from hydrogen utilizers such as *Desulfotomaculum* spp., *Desulfovibrio aespoensis* (both sulfate reducers), and *Acetobacterium carbinolicum* (an acetogen) suggest that carbon dioxide and hydrogen are main carbon and energy sources, respectively. No Archaea (including methanogens) were detected by culture or DNA [159]. The authors suggest that this is consistent with methanogens being outcompeted by sulfate reducers due to the sulfate concentration in the formation water.

In town gas storage (typically 45–60% hydrogen), significant hydrogen losses have been observed from stored gas, and both methanogenesis (resulting in volume loss) and microbial sulfate reduction (resulting in hydrogen sulfide production) were identified as concerns at a site at Lobodice in the Czech Republic [160]. Hydrogen sulfide production is also a concern for underground storage of hydrogen [161,162,163], particularly for storage in porous (e.g., sandstone) reservoirs [164]. Numerical modeling has highlighted the potential for microbial hydrogen consumption at the front where injected hydrogen displaces the existing gas. Depending on the environmental conditions, this could lead to methanogenesis or hydrogen sulfide production [165,166]. Note that the modeling showing microbial activity was based on storage in permeable reservoirs; storage in salt formations is likely to present a more challenging environment to organisms, but further research is required to rule out any possible microbial activities. 

Reviews of injection of hydrogen into underground methane storage (typically at 5–10% hydrogen) identify sulfate reduction, methanogenesis, and acetogenesis as the three main microbial reactions that could occur within a storage site, potentially even at temperatures up to 90 °C and at high salinities [163,167]. These reviews have recommended that individual assessments of sites before injection are required to establish the potential for microbial activity. Interestingly, laboratory studies carried out by Wurdemann et al. (reported in Reference [163]) did not observe any changes to gas composition or biofilm production in experiments containing 8% hydrogen unless carbon dioxide and nutrients were added. Nutrients from drilling fluids etc. have been reported to provide the nutrients/carbon to support microbial growth [168]. One innovative project aims to turn these microbial metabolisms that are often considered to have negative impacts, into a subsurface industrial process. The Underground Sun Conversion project is planning to inject hydrogen generated by renewable energy along with carbon dioxide from biomass burning into a gas reservoir, allowing methane formation to occur at over 1000 m below ground, providing a carbon-neutral methane supply that can be withdrawn when needed [169].

Microbiological activity has been identified as having potentially beneficial (e.g., enhancing sealing [170]) or negative impacts (e.g., blocking injection, corroding infrastructure [168,171]) on carbon dioxide injection or storage. As interest in a hydrogen economy grows, there will likely be an increased demand for carbon capture and storage (CCS) from methane steam reformation of methane to hydrogen. Carbon dioxide from this source is expected to contain some hydrogen and could increase microbial activities as observed in the co-storage of methane and hydrogen (e.g., Reference [160]). Several studies have been carried out looking at the reaction of microbial communities in response to carbon dioxide injection into potential reservoir materials or in pilot storage experiments; however, none, to the best of our knowledge, looked at the effect of hydrogen impurities. However, this topic is the focus of current work in the ERA-NET ELEGANCY project [172]. 

Investigations of sites proposed for carbon dioxide storage found representatives of genera that contain known hydrogenotrophs, including acetogens, sulfate reducers, *Hydrogenophaga*, *Acidovorax*, and *Ralstonia* spp. to be abundant [173,174]. Several laboratory and field studies showed increased levels of methanogens, especially in the initial phases of carbon dioxide injection. Following the start of injection of carbon dioxide, methanogenic archaea were seen to dominate the microbial community. Following a period of about five months, however, sulfate reducers were observed to outcompete the methanogens resulting in the precipitation of iron sulfide in the well and, subsequently, a reduction of injection rates [168,171]. A different response was reported at an injection into the Paaratte Formation of the Otway Basin, where a shift away from fermentative metabolism toward respiratory metabolisms was observed [174]. Here, an increase in methanogenesis in response to injected carbon dioxide has also been suggested [174]. In laboratory studies at elevated pressures, temperatures, and salinities, hydrogenotrophic methanogens were able to outcompete hydrogenotrophic sulfate reducers [175].

### 9.3. Radioactive Waste Disposal

Researchers investigating the safe disposal of radioactive waste in geological repositories have carried out a considerable amount of work to understand hydrogen generation from metal corrosion in the repository (e.g., of waste containers, spent fuel components, and its subsequent consumption) [152,176,177,178,179,180]. The main concerns with regard to hydrogen generation are the impact on the structural integrity of the repository, the potential for the hydrogen to act as a “carrier” for other radioactive gaseous species (e.g., ^222^Rn and ^14^C-bearing species such as CO_2_ and CH_4_) present within the waste inventory, and damage to the engineered barrier and adjacent host rock through over-pressuring. Under anaerobic conditions, corrosion of metal produces hydrogen by the reduction of water. Depending on the waste type, the metals present may include canister materials (e.g., carbon steel, stainless steel, or copper) and/or reactive metallic waste components (e.g., aluminum, Zircaloy cladding, Magnox cladding, and uranium). The rate of hydrogen production is limited by the availability of water, the surface area of the reacting metal, and the corrosion rate of each metal species [151,178,181]. The potential for this hydrogen to allow the growth of hydrogenotrophic iron and sulfate reducers has also received much attention because of the possibility of these processes causing further corrosion, altering clay barriers, or consuming excess hydrogen gas [11,182]. There is also some evidence to suggest that microbial precipitation of Cu_2_S films could slow corrosion by increasing resistance to attack by chloride ions [183]. However, the presence of sulfide can be considered deleterious since it is also known to enhance the corrosion rate of metallic copper [184]. Studies undertaken in several underground research laboratories in geologies relevant to radioactive waste disposal revealed the presence of native microbial communities heavily dependent on hydrogen metabolism [38,102,103,109]. Hydrogen injection experiments at the granitic Äspö Hard Rock Laboratory indicate hydrogen stimulates the microbial community, increasing microbial growth rates, and increasing sulfide and acetate production [185]. Ecological models have been developed for the native communities at the Horonobe Underground Research Laboratory [186] and for hydrogen-stimulated communities at the Mt Terri Underground Laboratory, Switzerland [187]. Both reveal a complex ecological network based on hydrogen metabolism. Under natural conditions, fermentation of organic matter in the sediment generates hydrogen, along with carbon dioxide and simple organic compounds that support a more diverse community, including heterotrophs. Microbial activities in clay environments such as Mt Terri are known to be low due to a combination of limited pore space, water, and organic carbon availability [188]. Microbial activity might be limited to fractures, which could be generated within a repository excavation-damaged zone. Increased hydrogen supply (as would be expected from canister corrosion) could stimulate a community of autotrophic hydrogen oxidizers. As biomass increases and cells eventually die, the necromass supports fermenters and, subsequently, heterotrophic sulfate reducers that oxidize fermentation products to carbon dioxide. The activity of sulfate reducers has been reported to increase corrosion of steel in repository conditions in clay host rock [189] and may alter the structural ferric iron in bentonite, with possible implications for its performance as a barrier material [190]. In an operational disposal facility, the complex processes involved in the evolution of any site-specific, repository environment will need to be taken into consideration to determine whether the timing of hydrogen release coincides with the appropriate environmental conditions (e.g., temperature, radiation, and pH) that are compatible with microbial activity and, therefore, whether or not this should be considered within the safety case. 

## 10. Summary and Future Research

Natural hydrogen gas occurs in various geological environments. This is of considerable interest to microbiologists investigating the limits of microbial life and the origins of life on Earth, or the potential for life on other planets. The main natural occurrences of hydrogen are gas seepages associated with the serpentinization of ultramafic rocks in ophiolite complexes. Other significant geological occurrences of hydrogen include hydrogen-rich gas reservoirs, radiolysis of water, and organic matter in crystalline basement rocks, and potassium-rich evaporites, halite deposits, and some hydrothermal vents and geothermal systems. Substantial evidence is being accumulated on the existence of microbial communities capable of utilizing and producing hydrogen in the subsurface. Hydrogen concentrations are often maintained at low levels due to microbial consumption processes, which may hide an active but cryptic hydrogen economy. Understanding these environments can provide an insight into engineered systems with elevated hydrogen concentrations. Due to the variety of these environments, a range of microbial responses are possible; however, some key themes can be drawn out. These themes may be of relevance to a number of subsurface industries where hydrogen is stored or where it is generated through natural processes or interactions between microbes and components of the engineered environment. Evidence from industry shows that many of the organisms that consume hydrogen are also implicated in the corrosion of metal infrastructure (with sulfate reducers being of particular concern). In addition to corrosion, other negative impacts of microorganisms that have been reported (e.g., reduction of gas injectivity in CCS projects) could also be increased by elevated hydrogen concentrations stimulating the microorganisms. The potential for methane production using hydrogen has been noted in some high-hydrogen environments. In some instances, this resulted in significant changes in stored gas volume; however, work is underway to exploit this natural process to generate methane in the subsurface from renewable energy and waste carbon dioxide.

The question of whether subsurface microbial ecosystems exist that are truly independent of photosynthesis remains to be fully answered. This is important because, if proven, it provides insights into a potential mechanism for how life developed on Earth, and is critical for assessing the potential for life to be supported on other planets. Demonstrating this independence remains challenging due to the difficulties associated with sampling from these deep environments, and with distinguishing between different sources of hydrogen. It is likely that additional site-specific experimental studies and modeling will be required to build a body of evidence to support or refute the SLiME hypothesis.

The thermodynamic data presented in the review are under standard conditions. Although these data are useful as a quick overview to compare the different hydrogen-cycling processes, the subsurface is not under ideal conditions. Using a range of geochemical data from subsurface environments, the controls on free energy could be used to predict likely dominant microbial process. Free energy calculations could be combined with additional variables (such as energetic cost of biosynthesis and metabolic strategy as considered in Reference [191]) to further understand microbial metabolism in these environments 

The frequency and distribution of sampling is an issue for those interested in industrial applications; in the context of radioactive waste disposal or CCS, the questions that need to be answered relate to processes that could occur over many thousands of years. In the absence of long-term data from these sites, and limited access to samples from operational sites, laboratory experimentation combined with modeling is one approach to understanding how microbial activities could impact on operations in the long term. Another approach could be to identify whether sites with naturally elevated hydrogen can act as analogs of particular aspects of the industrial operation of interest. Careful study of such sites could provide insights into how microbial communities might respond over longer periods than possible to study in the laboratory.

A further gap in the knowledge identified by this review is that it is difficult to describe in detail how exposure to hydrogen changes microbial community composition. While it is true that changes to communities can be described in terms of broad changes in functional groups (e.g., methanogens and sulfate reducers), describing how hydrogen exposure changes communities at finer scales (e.g., at the level of genera) is not really possible. While many of the studies in this review describe sample sites where hydrogen is generated, the differences in the community at different hydrogen concentrations were only reported in the Rio Tinto study [50]. This could be investigated by correlating microbial community composition to hydrogen concentration at sites within a particular geological setting, or by conducting laboratory experiments. Such research would provide information about the metabolic potential of communities at different hydrogen concentrations and would inform the potential for impact on industrial operations. In addition to reports of increased abundances of hydrogenotrophic sulfate reducers and methanogens, other more surprising organisms are sometimes identified. *Hydrogenophaga* spp. often appear as a common member of the microbial community in studies reported here. Little is known about the environmental role or significance of *Hydrogenophaga* in the subsurface. Since this genus is considered to be aerobic, the frequency with which they are reported in high abundance in typically anoxic subsurface environments is puzzling. Perhaps this is one area that deserves further investigation in order to assess what impacts they might have as a result of increased number and activity of this genus, and whether they are playing an important role in hydrogen cycling in the subsurface. Similarly, the report of hydrogen-consuming Cyanobacteria at the Rio Tinto site [192] is interesting as, although these organisms have previously been detected in the subsurface, this is the first evidence that they are active hydrogenotrophic members of the community. This poses questions relating to how widespread they are in the subsurface, their evolutionary origin, and relationship to other cyanobacteria. Nitrogen-fixing bacteria produce hydrogen, but their distribution and their activity in the subsurface has not been extensively studied. Given that some hypotheses of the deep microbial ecosystems of significance to radioactive waste disposal [193] assume nitrogen can be obtained via nitrogen fixation, it would be appropriate to investigate this process further in the subsurface. This would not only provide evidence for the ability of microbial communities to obtain nitrogen independently of photosynthesis in environments where there is no other microbially available nitrogen source, but would also determine whether or not microbial ecosystems in granitic settings around a radioactive waste disposal facility are likely to be nitrogen limited.

The constraints on subsurface microbial hydrogen cycling are starting to be understood; however, there is little information on hydrogen turnover rates in natural environments, or how they would react to elevated hydrogen concentrations. Such information would enable the fate of hydrogen in subsurface industrial settings to be better understood and the impact of microbial activity to be better predicted.

## Figures and Tables

**Table 1:** Abiotic processes generating hydrogen.

Process	Equation	Typical Geological Setting	References
Graphitization	CH_4 (g)_ → C + 2H_2 (g)_	Crystalline basement	[1,12]
Radiolysis of water	$2H_{2}O\overset{\mathrm{ionising}\;\mathrm{radiation}}{\to }H_{2}O_{2}+H_{2}$	Crystalline basement with high uranium, thorium, and potassium	[12,13]
Reactions of water with ^40^Ca daughter produced by radioactive decay of ^40^K	^40^Ca + 2H_2_O → Ca(OH)_2_ + H_2 (g,aq)_	Crystalline basement and potassium rich rocks (e.g., Kupferschiefer and Permian potash deposits at Stassfurt in Germany, Verkhmekamsk in Russia, and Boulby in the United Kingdom)	[1,14,15,16]
Serpentinization	e.g., olivine + water → serpentine + magnetite + hydrogen 6[(Mg_1.5_Fe_0.5_)SiO_4_] + 7H_2_O → 3[Mg_3_Si_2_O_5_(OH)_4_] + Fe_3_O_4_ + H_2_	Mafic and ultramafic rocks	[12,17,18,19,20]
Cataclasis	≡Si· + H_2_O → ≡Si + OH + H 2H → H_2_	Associated with silicate minerals in active fault zones	[21,22,23,24]

**Table 2:** Microbial hydrogen-generating reactions. Range of fermentation free energy of reactions from Reference [41], and adjusted to per mole hydrogen. Phosphite oxidation free energy from Reference [42]. All other free energies of reaction were calculated from thermodynamic data in Reference [43].

Hydrogen-Generating Process	Reaction	Free Energy ΔG^0′^ (kJ·mol^−1^ H_2_)
Fermentation	Multiple pathways that breakdown large organics into smaller organics, e.g., mixed acid fermentation e.g., C_6_H_12_O_6_ + 4H_2_O → 2CH_3_COO_3_^−^ + 2HCO_3_^−^ + 4H^+^ + 4H_2_	−54.0 to 24.2
Nitrogen fixation (nitrogenase activity)	N_2_ + 8H^+^ + 8e^−^ (Fd_red_) → 2NH_3_ + H_2_ (+Fd_ox_)	−18.1
Anaerobic carbon monoxide oxidation	CO + H_2_O → CO_2_ + H_2_	−19.9
Phosphite oxidation	H_3_PO_3_ + H_2_O → H_3_PO_4_ + H_2_	−46.3
Acetate oxidation	¼CH_3_COO^−^ + ¼H^+^ + ½H_2_O → H_2_ + ½CO_2_	23.7

Fd_red_ = reduced ferrodoxin, Fd_oc_ = oxidized ferrodoxin.

**Table 3:** Examples of microbial hydrogen-consuming reactions. Based on data in Reference [58]. Free energies not included in Reference [58] were calculated from Gibbs free energies of formation [42]. Hydrogen threshold values were expanded using provided references.

Hydrogen-Consuming Process	Reaction	Free Energy ΔG^0′^ (kJ·mol^−1^ H_2_)	Hydrogen Threshold (nM)	References
Hydrogenotrophic methanogenesis	¼HCO_3_^−^ + H_2_ + ¼H^+^ → ¼CH_4_ + ¾H_2_O	−33.9	0.4–95	[59]
Acetogenesis	½HCO_3_^−^ + H_2_ + ¼H^+^ → ¼CH3COO^−^ + 2H_2_O	−26.1	336–3640	[58]
Sulfate reduction	¼SO_4_^2−^ + H_2_ + ¼H^+^ → ¼HS^−^ + H_2_O	−38.0	1–15	[58]
Sulfur reduction	H_2_ + S → H_2_S	−33.1	2500	[58]
Iron(III) reduction	2FeOOH + H_2_ + 4H^+^ → 2Fe^2+^ + 4H_2_O	−228.3	0.1–0.8	[58]
Aerobic hydrogen oxidation (Knallgas)	H_2_ + ½O_2_ → H_2_O	−237	0.051	[55]
Dehalorespiration	Halogenated compounds + H_2_ → dehalogenated compounds + 2HCl	−230 to −187	0.27–2	[60]
Fumarate respiration	H_2_ + fumarate → succinate	−86.2	0.015	[58]
Denitrification	⅖NO_3_^−^ + H_2_ + ⅖H^+^ → ⅕N_2_ + 1⅕H_2_O	−240.1	<0.05–0.5	[61]
